# Association between the use of electronic cigarettes and myocardial infarction in U.S. adults

**DOI:** 10.1186/s12889-024-19561-z

**Published:** 2024-08-05

**Authors:** María José Farfán Bajaña, Juan Carlos Zevallos, Ivan Chérrez-Ojeda, Geovanny Alvarado, Tiffany Green, Betty Kirimi, Daniel Jaramillo, Miguel Felix, Emanuel Vanegas, Alejandra Farfan, Manuel Cadena-Vargas, Daniel Simancas-Racines, Marco Faytong-Haro

**Affiliations:** 1 School of Medicine, Universidad Espíritu Santo, Samborondón, Ecuador; 2 Respiralab Research Group, Guayaquil, Ecuador; 3 College of Medicine, American University of Antigua, Osbourn, Antigua and Barbuda; 4 Universidad Estatal de Milagro, Milagro, Ecuador; 5 Centro de Investigación en Salud Pública y Epidemiología Clínica (CISPEC), Facultad de Ciencias de la Salud Eugenio Espejo, Universidad UTE, Quito, 170527 Ecuador; 6 Universidad Católica Santiago de Guayaquil, Guayaquil, Ecuador; 7 Department of Internal Medicine, MetroWest Medical Center, Framingham, United States; 8 Department of Internal Medicine, NYC Health + Hospitals Woodhull, Brooklyn, United States

**Keywords:** E-cigarettes, Vaping, Myocardial infarction, Coronary heart disease, Adults

## Abstract

**Background:**

Compared with conventional cigarettes, electronic cigarettes are less harmful in some studies. However, recent research may indicate the opposite. This study aimed to determine whether e-cigarette use is related to myocardial health in adults in the U.S.

**Methods:**

This study used data from the 2020 Behavioral Risk Factor Surveillance System (BRFSS), a cross-sectional survey of adult US residents aged 18 years or older. We examined whether e-cigarette use was related to myocardial infarction byapplying a logistic regression model to calculate odds ratios (ORs) and 95% confidence intervals (CIs).

**Results:**

The final analytical sample included 198,530 adults in the U.S. Logistic regression indicated that U.S. adults who reported being former and some days of e-cigarette use had 23% and 52% greater odds of ever having an MI, respectively, than did those who reported never using e-cigarettes (OR = 1.23, 95% CI 1.08–1.40, *p* = 0.001; OR = 1.52, 95% CI 1.10–2.09, *p* = 0.010).

**Conclusions:**

The results suggest that former and someday users of e-cigarettes probably have increased odds of myocardial infarction in adults in the U.S. Further research is needed, including long-term follow-up studies on e-cigarettes, since it is still unknown whether they should be discouraged.

## Introduction

Cardiovascular disease (CVD) is the leading cause of death in the United States (U.S.). Approximately 17.9 million individuals die annually from CVD, according to the World Health Organization (WHO) [1]. It is estimated that approximately 363 billion dollars are spent each year on health care, pharmaceuticals, and lost productivity due to cardiovascular diseases in the United States. Coronary heart disease (CHD) is the most common CVD. Approximately 18.2 million adults over 20 years of age have CHD (6.7%).

Coronary heart disease (CHD) can cause myocardial infarction (MI), affecting 805,000 people annually in the U.S., leading to irreversible cardiac muscle damage due to decreased blood flow and hypoxemia [2]. According to the American Heart Association (AHA), approximately 800,000 cardiovascular deaths occur every year, and nearly 20% of these deaths are due to cigarette smoking [3]. In 2019, approximately 4.5% of the adult population in the United States were current (e-cigarette) users [4]. E-cigarettes, or “electronic nicotine delivery systems,” are recreational devices powered by a lithium battery that vaporize interchangeable liquid cartridges added to the vaporization chamber. Common ingredients in e-cigarettes include ultrafine particles, nicotine, toxic gases, propylene glycol, glycerin, and flavoring chemicals. Additionally, some authors have reported the presence of contaminants such as diacetyl, volatile organic compounds, heavy metals, acetone, benzaldehyde, siloxanes, reactive oxygen species, polycyclic aromatic hydrocarbons, tobacco-specific nitrosamines, fungal glucans, and bacterial endotoxins [5, 6]. Even if devices are labeled as having “no nicotine,” many of them contain it to a high degree [7].

In a systematic review, researchers found that e-cigarette users are 1.33 times more likely to suffer an MI [8]. There have also been documented cardiovascular effects associated with E.C.s, including nicotine-induced sympathetic activity, tachycardia, increased blood pressure, cardiac output, myocardial oxygen consumption, and vasoconstriction of cutaneous and coronary blood vessels [6]. These cardiovascular effects may be due to the suggested arrhythmogenic properties of nicotine, reactive oxygen species, free radicals, carbonyl production, and particulate matter delivery [9]. Consequently, it may lead to inflammation, endothelial dysfunction, lipid oxidation, and thrombosis [10]. Based on data collected from the National Health Interview Surveys (NHIS) of 2014 and 2016, Alzahrani et al. found that e-cigarette users were associated with an increased risk of myocardial infarction (OR = 1.79, 95% CI = 1.20, 2.66; *p* = 0.004), even though former and occasional e-cigarette users did not appear to have a significant association with this outcome [11]. In contrast, Goniewicz et al. concluded that there was no difference between e-cigarette users and non-users in cardiovascular outcomes (including stroke, myocardial infarction, and coronary heart disease) [12]. In 2016–2017, approximately 3.5–4.5% of the population in the U.S. used e-cigarettes [10]. Using data from the BRFSS, Osei et al. found that people who used both combustible cigarettes and e-cigarettes had 36% greater odds of cardiovascular disease [13–15]. As the use of e-cigarettes has increased significantly during the ongoing COVID-19 pandemic [16, 17], more research is needed to determine whether e-cigarettes contribute to MI or CHD. We used the BRFSS dataset from 2020, one of the most critical pandemic years, to study the association between e-cigarette use and myocardial infarction in adults in the United States. To our knowledge, this is one of the first studies to test the correlation between e-cigarette use and the frequency of MI/CHD during the pandemic.

## Materials and methods

### Setting

For this analysis, we used the BRFSS, a national United States cross-sectional health-related telephone survey implemented employing RDD (random digit dialing). Since 1964, this survey has collected self-reported data about U.S. residents’ health habits and chronic conditions and their use of preventive services. For this analysis, we chose 2020, a year in which e-cigarette use increased and many residents were in lockdown due to the COVID-19 pandemic [18, 19]. Assisted by the CDC, state health departments and different research institutes conducted a cross-sectional telephone survey over landline and cellular telephones using a standardized methodology and questionnaire. Each year, the core questionnaire includes questions about emerging or “late-breaking” health issues, with e-cigarettes being one of the latest issues addressed. The sampling frame of the household survey included all adults aged 18 years or older who lived in private residences or college housing at the time of the study and persons on residential phone lines at the time of the survey. The median response rate for all states and territories was 47.9%. The study area is representative of 50 states, the District of Columbia, and three U.S. territories [2].

### Analytical sample

For 2020, the initial survey sample comprised 401,958 individuals; however, 264,561 individuals were randomly selected to answer the optional core questionnaire about e-cigarettes. We excluded 2,313 patients who were missing answers to the question about ever-reported myocardial infarction, our dependent variable. In addition, 715 patients were excluded because they did not know or refused to answer the question about e-cigarette use, which was our independent variable. We then excluded 69 patients who were missing data for the e-cigarette frequency variable. Finally, if participants had missing information on any control variables, they were removed from the sample, resulting in a final analytical sample size of 198,530.

### Variables

#### Outcome variables

Our outcome variable was whether the respondent had myocardial infarction (MI) or CHD. They were assessed using the following items: “Has a doctor, nurse, or another health professional ever said that you had a heart attack, also known as MI?” and “Has a doctor, nurse, or other health professional said you had angina or coronary heart disease?”. The answers to either question were “Yes, No, Don’t know/Not sure, and Refused.” Our outcome variable was appraised as follows: 1, reported having MI or CHD; 0, did not report MI or CHD.

#### Independent variables

In the CDC BRFSS 2020 survey, participants self-reported their electronic cigarette use, which was measured and assessed by two main questions [2]. The first question asked if they had ever used e-cigarettes or other electronic vaping products, even once, in their entire life, with a dichotomous answer (yes/no). The second question characterized the frequency of current e-cigarettes and other e-vapping products. The answers were ordinal: “Every day” (1), “Some days” (2), and “Not at all” (3). We recoded both questions into one primary variable reflecting their current and past e-cigarette use. First, if they replied to the first question that they had never used an e-cigarette, not even once, we recoded them as “Never.” Second, those who responded that they had ever used electronic cigarettes and reported not using them at all were now recoded as “Former” users. If they reported smoking e-cigarettes “some days” or “every day” in the second question, they were left unchanged.

#### Confounding variables

The following confounding variables were included and assessed: age, sex, race/ethnicity, marital status, tobacco smoking status, alcohol intake, education level, diabetes mellitus, income level, physical activity, and BMI [5, 16, 17, 20–27].

For age, from 12 categories of the original survey (“Age 18 to 24”, “Age 25 to 29”, “Age 30 to 34”, “Age 35 to 39”, “Age 40 to 44”, “Age 45 to 49”, “Age 50 to 54”, “Age 55 to 59”, “Age 60 to 64”, “Age 65 to 69”, “Age 70 to 74”, “Age 80 and older”), we recoded it as 5 (1: Age 18 to 34, 2: Age 35 to 49, 3: Age 50 to 64, 4: Age 65 to 79, 5: Age 80 or older).

For education level, from 6 original items [“Never attended school or only kindergarten,” “grades 1–8 (elementary),” “grades 9–11 (some high school),” “grade 12 or GED (high school graduate),” “college one year to three years (Some college or technical school),” “college four years or more (College graduate)]], we recoded them into “Never attended school or only kindergarten,” “grades 1–11/12 or GED (Elementary and high school)”, and “college 1–4 years or more/graduate”.

Alcohol consumption was a constructed variable obtained from (1) the total number of alcoholic beverages consumed per week (by multiplying the total number of drinks per day by the average number of drinks consumed per occasion for seven days) and (2) self-reports of having consumed at least one alcoholic beverage in the past 30 days. Final alcohol consumption was calculated as follows: Yes/No.

Income levels were assessed as follows: “less than 10,000,” “less than 15,000,” “less than 20,000,” “less than 25 000,” “less than 35 000,” “less than 50 000,” “less than 75,000” and “75,000 or more.” However, for simplicity, after recodification, they ended up “<25,000”, “25,000–49,999”, “50,000 to 74,999” and “>75,000”.

Diabetes mellitus was assessed using the following question: Have you ever been told you had diabetes? (If “Yes” ´ and the respondent is female, it was asked: Was this only when you were pregnant? ). The possible answers were “Yes,” “Yes, but female said only during pregnancy,” “No,” and “No prediabetes or borderline diabetes.” This variable was recoded as follows: “Yes,” “Yes, but female told only during pregnancy,” and “No or no prediabetes or borderline diabetes.”

Exercise level was assessed by the following question: “During the past month, other than your regular job, did you participate in any physical activities or exercises such as running, calisthenics, golf, gardening, or walking for exercise?” The variables consisted of yes/no.

The questions “Do you now smoke cigarettes every day, some days, or not at all?” and “Have you ever used an e-cigarette or other electronic vaping product, even just one time, in your entire life?” were answered as “Every day,” “Some days,” “Not at all,” and “Yes,” “No,” respectively. Both questions were utilized to calculate a new variable categorizing smoker status into four levels: “Every day smoker,” “Some days smoker,” “Former smoker,” and “Nonsmoker.”

Race/ethnicity was assessed as “White,” “non-Hispanic,” “Black, Non-Hispanic,” “Asian,” “Non-Hispanic,” “American Indian/Alaskan Native, Non-Hispanic,” “Hispanic,” or “Other race, non-Hispanic.” Body mass index (BMI) was calculated from the original BMI variable obtained from the self-reported height and weight of the participants. It has four categories: underweight, normal weight, overweight, and obese. The sex variables were assessed as “Male” and “Female”.

### Statistical analysis

Before conducting the primary analysis, we examined the frequency distribution of the variables of interest for the entire analytical sample, for participants who had MI or CHD, and for those who did not. Table 1 shows the percentage levels of all explanatory and confounding variables in the analysis. Differences across groups were tested using the chi-squared test.

Logistic regression was performed to test the association between electronic cigarette use and the occurrence of MI or CHD. Table 2 shows the odds ratios and p values of the main explanatory variables and controls. We employed the corresponding weights for all analyses following the BRFSS guidelines [2]. BRFSS sampling weights were based on iterative proportional fitting or ranking to known proportions of age, sex, categories of ethnicity, geographic regions within states, marital status, education level, home ownership, and phone ownership. *The* STATA statistical software package version 17 was used for data cleaning and analysis.

### Ethical considerations

We evaluated the necessity of IRB (institutional review board) approval according to the FIU’s Institutional Review Board guidelines. According to their guidelines, this project did not require IRB approval because there was no direct interaction with human subjects, and the dataset was available in the public domain. We used the 2020 BRFSS dataset administered by the CDC for this analysis. Interested researchers can access public data via the following website: “https://www.cdc.gov/brfss/annual_data/annual_2020.html.” Other researchers will be able to access the dataset in the same manner as the author. The CDC required informed consent from all participants of the survey they administered. In addition, according to Article 43, Letter b of the Regulation for Approval and Monitoring of the Ethics Committee of Human Beings of Ecuador states that investigations using open or public data are considered risk-free.

## Results

### Descriptive and bivariate analysis

A total of 198,530 U.S. adults were included in the final sample. Over half (50.4%) of the respondents were male, and 49.6% were female. The differences between those who had MI or CHD and those who did not for all variables were statistically significant based on chi-square analyses (*p* < 0.005). Table 1 shows the descriptive results for all the variables of our analytical sample. Among reported cases of MI/CHD, males accounted for 62.14% and females for 37.86%. Of the total sample, 15% were attributed to former users. Married participants accounted for approximately half of the reported MI/CHD cases (52.64%). Approximately 77.6% of individuals with MI/CHD were white non-Hispanic individuals. Participants who had an education of some kind (high school/elementary or some college/college) reported the majority of MI occurrences (49.14% and 50.70%, respectively). The highest percentage of individuals with MI (36.32%) were reported at the lowest income level. A total of 38.64% of the participants with MI/CHD reported not exercising, and 77.84% of those without MI/CHD reported exercising. Compared to traditional smokers, *41.82% of those with MI/CHD were former smokers*. 61% of those with MI/CHD reported drinking alcohol in the past 30 days. Of the patients who reported MI or CHD, 20.81% were of normal weight, and 40.97% were obese. Finally, among the patients with MI/CHD, 33.56% had diabetes.

**Table 1:** Baseline characteristics of U.S. adults who responded to the 2020 BRFSS survey and who had myocardial infarction and coronary heart disease

	Total	Have not had MI or CHD	Have had MI or CHD	*p* value*
**Myocardial infarction or coronary heart disease**				-
Yes	7.21%	-	-	
No	92.79%	-	-	
**E-cigarette use**				< 0.001
Never	74.77%	74.29%	80.97%	
Former	19.69%	20.00%	15.68%	
Some days	3.07%	3.15%	2.04%	
Every day	2.46%	2.55%	1.31%	
**Sex**				< 0.001
Male	50.40%	49.49%	62.14%	
Female	49.60%	50.51%	37.86%	
**Age**				< 0.001
Age 18 to 34	28.08%	3.24%	26.29%	
Age 35 to 49	25.52%	9.02%	24.33%	
Age 50 to 64	26.63%	30.40%	26.90%	
Age 65 to 79	16.05%	42.46%	17.95%	
Age 80 or older	3.72%	14.89%	4.53%	
**Marital status**				< 0.001
Married	52.25%	52.21%	52.64%	
Divorced/Widowed/Separated	20.98%	19.72%	37.25%	
Never married/Unmarried couple	26.77%	28.07%	10.11%	
**Race/ethnicity**				< 0.001
White, Non-Hispanic	67.22%	66.41%	77.60%	
Black, Non-Hispanic	12.97%	13.17%	10.49%	
Asian, Non Hispanic	3.50%	3.67%	1.30%	
American Indian/Alaskan Native, Non-Hispanic	0.95%	0.92%	1.25%	
Hispanic	13.38%	13.82%	7.64%	
Other race, Non-Hispanic	1.99%	2.01%	1.72%	
**Education level**				< 0.001
Never attended	0.15%	0.15%	0.17%	
Up to high school/Elementary	39.13%	38.35%	49.14%	
Some college/College	60.72%	61.50%	50.70%	
**Income level**				< 0.001
<$25K	23.82%	22.85%	36.32%	
$25,000-$49,999	22.94%	22.65%	26.69%	
$50,000-$74,000	16.10%	16.20%	14.81%	
>$75K	37.14%	38.30%	22.18%	
**Exercise (in the past 30 days)**				< 0.001
Yes	76.65%	77.84%	61.36%	
No	23.35%	22.16%	38.64%	
**Traditional smoking**				< 0.001
Current smoker – now smokes everyday	11.67%	11.43%	14.77%	
Current smoker – now smokes somedays	4.70%	4.69%	4.85%	
Former smoker	25.30%	24.02%	41.82%	
Never smoked	58.33%	59.86%	38.56%	
**Alcohol consumption (in the past 30 days)**				< 0.001
Yes	53.59%	54.74%	38.86%	
No	46.41%	45.26%	61.14%	
**Body mass index (BMI)**				< 0.001
Underweight	1.59%	1.59%	1.59%	
Normal Weight	28.97%	29.61%	20.81%	
Overweight	34.82%	34.68%	36.63%	
Obese	34.61%	34.12%	40.97%	
**Diabetes**				< 0.001
Yes	12.03%	10.36%	33.56%	
Yes, gestational	0.89%	0.93%	0.29%	
No	87.08%	88.71%	66.15%	

*The significance of differences between the MI/CHD groups was calculated using chi-square tests

### Multivariate analysis

Logistic regression analysis was used to examine the impact of e-cigarettes on MI/CHD. We adjusted for sex, age, marital status, race/ethnicity, education level, income level, exercise, traditional smoking, alcohol use, BMI, and diabetes. Some days and former users of e-cigarettes had 52% and 23% greater odds of having an MI/CHD, respectively, than did those who reported never using them (OR = 1.23, 95% CI 1.08–1.40, *p* = 0.001; OR = 1.52, 95% CI 1.10–2.09, *p* = 0.010). Females were less likely to report having an MI/CHD (OR = 0.50, 95% CI 0.46–0.54; *p* = 0.000) than males were. Divorced/widowed/separated individuals were 37% more likely (OR = 1.37, 95% CI 1.19–1.58, *p* = 0.000) to have an MI/CHD than those who were not married. Regarding race, black non-Hispanics were 29% less likely (OR = 0.71, 95% CI 0.62–0.80, *p* = 0.000), and Hispanics were 37% less likely (OR = 0.63, 95% CI 0.49–0.81, *p* = 0.000) to report ever having an MI/CHD than White non-Hispanics. As the participants’ age, level of income, and BMI increased, they had greater odds of reporting MI/CHD. Those who exercised had lower odds of ever reporting having an MI/CHD (OR = 0.73 95% CI (0.68–0.80), *p* = 0.000) than those who did not. The results showed that, from being a former traditional smoker to being an every day traditional smoker, the odds of having an MI/CHD increase. On the other hand, having diabetes almost doubled the odds of reporting ever having an MI/CHD (OR = 2.08 95% CI 1.90, 2.27, *p* = 0.000). Finally, in comparison to their reference categories, no significant association was found between having an MI/CHD and being every day users of e-cigarettes, being American Indian/Alaskan Native Non-Hispanic, having up to high school/elementary level of education, or having some college/college education.

**Table 2:** Logistic regression analysis of adults who responded to the 2020 BRFSS. The outcome variable was myocardial infarction/coronary heart disease

	OR	Have had MI or CHD	
		CI	*p* value
**E-cigarette use (Reference never)**			
Former	1.235***	(1.0876–1.4017)	0.001
Some days	1.524***	(1.1078–2.0976)	0.010
Every day	1.085	(0.7758–1.5172)	0.634
**Sex (Reference = Male)**			
Female	0.508***	(0.4694–0.5496)	< 0.01
**Age (Reference = Age 18 to 34)**			
35 to 49	2.645***	(2.0444–3.4211)	< 0.01
50 to 64	7.104***	(5.6512–8.9308)	< 0.01
65 to 79	14.37***	(11.3194–18.2516)	< 0.01
80 or older	20.78***	(16.1018–26.8089)	< 0.01
**Marital status (Reference = Never married)**			
Married	1.213***	(1.0549–1.3936)	0.007
Divorced/Widowed/Separated	1.373***	(1.1925–1.5802)	< 0.01
**Race/ethnicity (Reference = White**,** Non-Hispanic)**			
Black, Non-Hispanic	0.711***	(0.6252–0.8092)	< 0.01
Asian, Non Hispanic	0.555***	(0.4094–0.7524)	< 0.01
American Indian/Alaskan Native, Non-Hispanic	1.036	(0.8244–1.3023)	0.760
Hispanic	0.635***	(0.4927–0.8184)	< 0.01
Other race, Non-Hispanic	0.866	(0.7281–1.0289)	0.102
**Level of education (Reference = Never attended)**			
Up to high school/Elementary	0.986	(0.3883–2.5033)	0.976
Some college/College	0.944	(0.3705–2.4048)	0.904
**Income level (Reference=<$25K)**			
$25,000-$49,999	0.763***	(0.6859–0.8479)	< 0.01
$50,000-$74,000	0.684***	(0.6071–0.7716)	< 0.01
>$75K	0.537***	(0.4787–0.6022)	< 0.01
**Exercise (in the past 30 days) (Reference = No)**			
Yes	0.738***	(0.6804–0.8011)	< 0.01
**Traditional Smoking (Reference = Never smoked)**			
Current smoker - now smokes every day	1.538***	(1.3500- 1.7532)	< 0.01
Current smoker - now smokes some days	1.592***	(1.3391–1.8921)	< 0.01
Former smoker	1.564***	(1.4298–1.7098)	< 0.01
**Alcohol use (in the past 30 days)(Reference = No)**			
Yes	0.757***	(0.6978–0.8210)	< 0.01
**Body mass index (BMI) (Reference = Underweight)**			
Normal Weight	0.795	(0.5724–1.1028)	0.169
Overweight	0.956	(0.6897–1.3241)	0.785
Obese	1.064	(0.7688–1.4714)	0.710
**Diabetes (Reference = No)**			
Yes	2.081***	(1.9061–2.2722)	< 0.01
Yes, gestational	0.775	(0.4176–1.4389)	0.420
Constant	0.0207***	(0.0076–0.0562)	< 0.01
Observations	198,530		

The confidence intervals are in parentheses

*** *p* < 0.01, ** *p* < 0.05, * *p* < 0.1

Coefficients are represented as odds ratios (ORs)

## Discussion

Tobacco smoking declined by 8.4% from 2005 to 2020, and surprisingly, e-cigarette use increased by 9.9% from 2017 to 2020 in the U.S. [4, 28]. According to Gallus et al., e-cigarette usage increased from 8.1 to 9.1%, and traditional cigarette usage increased from 4.0 to 4.5% during the pandemic, suggesting an e-cigarette preference [18]. Several factors may contribute to consumers choosing e-cigarettes, such as their appeal, impact on social media, low cost, ease of finding, accessibility, use, flavorfulness, and convenience. Studies have also shown that they have health benefits and are effective in helping traditional smokers quit smoking [29]. Among the participants in our study, 19.69%, 2.46%, and 3.07% reported being former, every day and some days e-cigarettes users, respectively. Also, our results showed 25.30%, 11.67%, and 4.7% participants reported being former, daily and some days traditional smokers which corresponds of 41.67% of the total sample. The elevated number of traditional smokers in our study could be explained by the fact that our population mainly comprised adults who preferred traditional smoking, as one study revealed that young adults aged 18–24 years were significantly more likely to use e-cigarettes than adults aged 25 years and older [30]. Our findings suggest that the likelihood of MI/CHD increases with age and income. Older adults are more likely to suffer from most types of CVD than other age groups are [31].

However, a greater risk of adverse health outcomes such as myocardial infarction may be associated with low income, according to Coughlin and Young [32]. In our study, those who were divorced, widowed, or separated from their spouses were 37% more likely to develop MI/CHD than were those who had never been married. There are possible explanations for this phenomenon, such as the possibility of divorce or marital separation causing substantial psychological distress, which sometimes leads to cigarette smoking as a coping mechanism [33].

In recent years, there has been some evidence of harm due to e-cigarettes, and biological investigations have supported these findings [34]. Among cardiovascular effects, e-cigarettes may increase blood pressure and heart rate after short-term exposure (5–30 min). Nicotine, a significant component of e-cigarettes, has been reported to cause endothelial dysfunction and vascular calcification in vascular walls and interfere with the growth of vessels after long-term exposure [35]. An injury model using the ferric chloride carotid artery showed a threefold reduction in occlusion time following nicotine exposure [36]. Moreover, a case‒control study reported increased levels of oxidative stress and a shift in cardiac autonomic balance toward sympathetic predominance after nicotine exposure [37]. A systematic review suggested that e-cigarette use causes cardiovascular harm by increasing the risk of thrombosis and atherosclerosis [38].

The effects of e-cigarettes are primarily associated with nicotine exposure. Flavoring chemicals, such as diacetyl (DA), also play an important role. A recent study reported that DA is associated with oxidative stress and inflammatory processes [39]. The effects of toxic components on e-cigarettes have been previously described. It is crucial to consider that there is a wide range of e-cigarettes owing to the different nicotine concentrations, volumes of e-liquid per product, carrier compounds, additives, and battery voltages. According to previous research, different combinations of these elements could cause variations in harm to the cardiovascular system [40, 41]. Pharmacological studies on e-cigarettes have reported that nicotine is delivered more slowly than regular cigarettes [42]. Nevertheless, recent research has suggested that nicotine levels depend on the generation and consumption of e-cigarettes. Therefore, not all consumers experience the same amount of toxicity [6]. Unfortunately, there is uncertainty regarding whether device variability could change the results because they were not assessed.

Our results showed that using an e-cigarette at least once in life had 23% greater odds (OR = 1.23, 95% CI 1.08–1.40, *p* = 0.001), and using e-cigarettes for some days increased the likelihood of ever reporting having an MI/CHD by 52% (OR = 1.52, 95% CI 1.10–2.09, *p* = 0.010) in comparison to never using them. Similar to the CDC 2016 BRFSS study, e-cigarette users were found to have a significantly greater risk of angina and/or coronary heart disease (OR, 1.4; 95% CI, 1.35–1.46) and MI (OR, 1.59; 95% CI, 1.53–1.66) than nonusers [43]. Moreover, Vindhyal et al. reported NHIS results and concluded that e-cigarette users had 50% greater odds of developing MI (OR, 1.56; 95% CI, 1.45–1.68) [14]. In contrast to our results, two large cross-sectional studies reported no significant association between exclusive e-cigarette use and CVD [14, 44]. Additionally, our study showed that as the frequency of use increased (from former to some-day users), the likelihood increased, in contrast to the findings of Alzahrani et al., who reported that there was no significant increase in the odds of MI risk for former or some-day e-cigarette users [45]. This author reported that daily use of e-cigarettes was 1.70 times greater than that of healthy nonusers, whereas in our study, no significant association was found between every day users and nonusers.

An intriguing aspect of our findings is the lack of statistical significance when comparing daily e-cigarette users with nonusers in terms of MI/CHD risk. Several factors may have contributed to this finding. First, the number of participants categorized as daily e-cigarette users may have been too small to detect a statistically significant association with MI or CHD. Small sample sizes often lack the power to identify moderate or even large differences in risk, which could lead to a type II error (failing to detect an effect that is present). Moreover, many daily e-cigarette users tend to be younger adults [46]. Younger individuals generally have a lower baseline risk of cardiovascular events such as MI and CHD [47]. The relatively short duration of e-cigarette use among this group might not be sufficient to manifest the long-term cardiovascular consequences [48]. This younger demographic may not have accumulated enough cumulative exposure to e-cigarette components to exhibit significant clinical outcomes at this stage [49]. The pathogenesis of cardiovascular diseases, including MI and CHD, often involves long-term exposure to risk factors that lead to cumulative epigenetic changes [50]. These changes can influence gene expression and contribute to the development of atherosclerosis and other cardiovascular conditions [51]. In younger e-cigarette users, the duration of exposure may not yet be sufficient for these epigenetic changes to result in overt clinical consequences [52]. Given these considerations, we recommend cautious interpretation and further investigation to elucidate this unexpected trend. To conclusively determine the cardiovascular risks associated with daily e-cigarette use, further research with larger sample sizes, longer follow-up periods, and careful consideration of confounding factors is needed. This research should include diverse populations, longer duration of exposure, and detailed assessments of both the types and usage patterns of e-cigarettes.

Nevertheless, we should keep in mind that e-cigarettes can be harmful to the cardiovascular system regardless of whether they are abused, as former e-cigarette users are likely to have consumed them at least once. Additionally, given the potential cardiovascular risks associated with e-cigarette use, it is imperative for public health policies to manage regular cardiovascular monitoring in users. Such proactive assessments can facilitate early detection of heart abnormalities, potentially preventing severe complications. By integrating this into policy, governments can underscore the health implications of e-cigarettes, prioritize prevention, and offset substantial healthcare costs associated with treating e-cigarette-induced cardiovascular issues in the long run [53].

## Limitations and recommendations

Traditional tobacco and its interaction with e-cigarettes were not evaluated entirely because of the way the questions were conducted, which did not allow causal ordering or permit the identification of interactions. It is still unclear whether recreational e-cigarettes are entirely safe in the long term for cardiovascular health, and the discouragement of e-cigarette use remains an option. Since this study was cross-sectional, prevalence bias was expected, and it was not possible to infer causality. Despite adjusting for age in our multivariate analysis, there remains a potential limitation related to causality bias. E-cigarette use is more prevalent among younger individuals compared to older adults. While we have accounted for age statistically, the demographic distribution of e-cigarette users could still influence the interpretation of causal relationships observed in our study. It’s important to recognize that factors associated with younger age groups, beyond age itself, may contribute to the outcomes studied. Future research should further investigate these associations to better understand the nuanced effects of e-cigarette use across different demographic groups. In addition, we relied on self-reported recall of MI/CHD patients, which may have caused self-report bias and impacted the accuracy of the results. Ideally, confirmative diagnostic tools such as angiography or laboratories should be implemented in future research. Hypertension and COVID-19 infection were not included as confounding variables, and since hypertension is one of the most important cardiovascular risk factors and since COVID-19 is an outbreak disease of the year the study was conducted, its exclusion is one of the limitations of this study.

Moreover, not every U.S. state was included in the exposure assessment questionnaire (BRFSS survey). Cohort studies have examined the cardiovascular system response to extended exposure to e-cigarettes. In addition, it is necessary to objectively quantify the amount of e-cigarette consumption in future research. Asking participants if their products are labeled nicotine containers should be relevant for future investigations. In the absence of information about the type or extent of myocardial infarction or the order of presentation between MI/CHD and the use of e-cigarettes, we cannot assess whether the exposure occurred before or after the outcome; therefore, we cannot determine the temporal association between them.

## Conclusions

Being a former or occasional user of an e-cigarette may increase their odds of developing an MI/CHD. Further research into the association between the use of e-cigarettes and MI/CHD should be conducted with higher and longer-term exposure to them, as well as investigating information regarding the type and quantity of e-cigarettes as well as the time, extent, and type of MI/CHD presented.

## Data Availability

The datasets analyzed during the current study are available in the 2020 Behavioral Risk Factor Surveillance System (BRFSS) repository administered by the CDC. Interested researchers can access public data via the following website: “https://www.cdc.gov/brfss/annual_data/annual_2020.html.” Other researchers will be able to access the dataset in the same manner as the author.
